# Hyperspectral data processing improves PpIX contrast during fluorescence guided surgery of human brain tumors

**DOI:** 10.1038/s41598-017-09727-8

**Published:** 2017-08-25

**Authors:** J. J. Bravo, J. D. Olson, S. C. Davis, D. W. Roberts, K. D. Paulsen, S. C. Kanick

**Affiliations:** 1 Thayer School of Engineering, Dartmouth College, Hanover, NH 03755 USA; 2 Norris Cotton Cancer Center, Dartmouth-Hitchcock Medical Center, Lebanon, New Hampshire 03756 USA; 3 Geisel School of Medicine at Dartmouth, Hanover, NH 03755 USA; 4 Section of Neurosurgery, Dartmouth-Hitchcock Medical Center, Lebanon, New Hampshire 03756 USA

## Abstract

Fluorescence guided surgery (FGS) using aminolevulinic-acid (ALA) induced protoporphyrin IX (PpIX) provides intraoperative visual contrast between normal and malignant tissue during resection of high grade gliomas. However, maps of the PpIX biodistribution within the surgical field based on either visual perception or the raw fluorescence emissions can be masked by background signals or distorted by variations in tissue optical properties. This study evaluates the impact of algorithmic processing of hyperspectral imaging acquisitions on the sensitivity and contrast of PpIX maps. Measurements in tissue-simulating phantoms showed that (I) spectral fitting enhanced PpIX sensitivity compared with visible or integrated fluorescence, (II) confidence-filtering automatically determined the lower limit of detection based on the strength of the PpIX spectral signature in the collected emission spectrum (0.014–0.041 μg/ml in phantoms), and (III) optical-property corrected PpIX estimates were more highly correlated with independent probe measurements (r = 0.98) than with spectral fitting alone (r = 0.91) or integrated fluorescence (r = 0.82). Application to *in vivo* case examples from clinical neurosurgeries revealed changes to the localization and contrast of PpIX maps, making concentrations accessible that were not visually apparent. Adoption of these methods has the potential to maintain sensitive and accurate visualization of PpIX contrast over the course of surgery.

## Introduction

Resection decisions made by neurosurgeons often involve trade-offs between maximizing tumor removal and minimizing function deficits secondary to surgery. The current state-of-the-art relies on visual inspection of the surgical field, assisted by image-guidance based on preoperative radiologic imaging. Fluorescence guided surgery (FGS) using aminolevulinic acid (ALA) induced protoporphyrin IX (PpIX) is a newer approach that offers immediate and concurrent intraoperative imaging for guiding resection decisions^1, 2^. The procedure involves administration of ALA, which is a non-fluorescent pro-drug that leads to a temporarily-enhanced accumulation of the endogenous fluorophore PpIX in malignant tissue. PpIX selectivity is based on a combination of pathophysiological hallmarks of cancer, including decreased vascular integrity and altered cellular metabolism^3^. However, standard FGS techniques rely on raw PpIX fluorescence from the surgical field, which may suboptimally reflect malignancy for two primary reasons. First, the raw fluorescence emissions contain background contributions from other sources (e.g. excitation light leakage, autofluorescence and photoproducts) which may alter the lower limit of sensitivity to PpIX^4^, and affect discrimination of locations having different PpIX accumulations. Second, raw fluorescence intensity is influenced not only by the concentration of PpIX but also by tissue optical absorption and scattering at excitation and emission wavelengths, which substantially distort the remitted signal^5, 6^. These distortions may be most critical near the end of surgery, when remaining disease must be identified within a surgical cavity. These factors represent important limitations of FGS based solely on using raw fluorescence emissions to identify residual tumor near the margins of the planned resection volume and/or as surgery approaches areas of critical brain function where resection decisions become more difficult.

**Figure 1:**
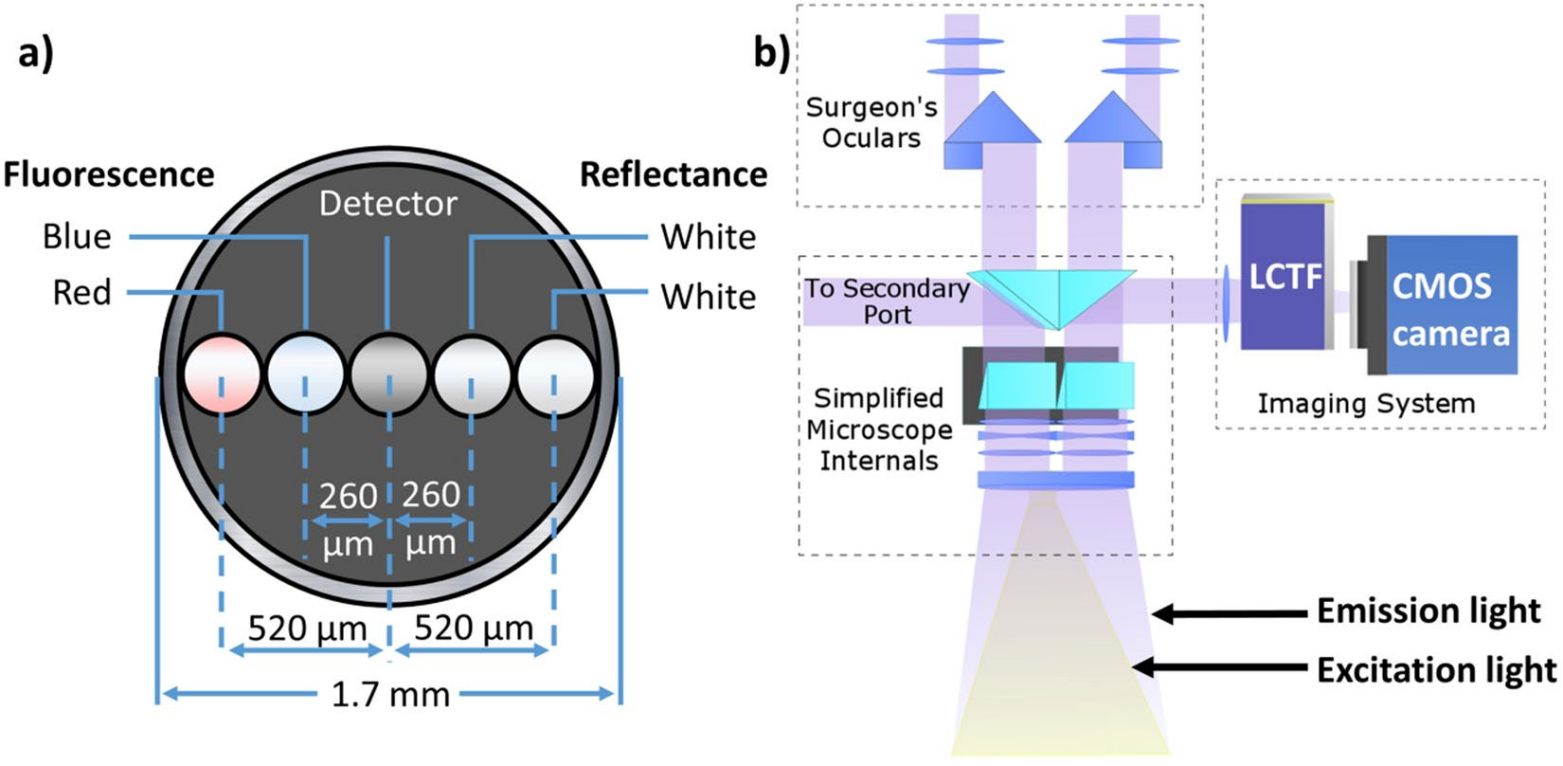
(**a**) Diagram of fiber arrangement on probe tip. White-light fibers used for reflectance are located 260 μm and 520 μm from the detector. The blue-light (405 nm) fiber used to excite PpIX was also located 260 μm from the detector. Red (635 nm) light data was not used for this study. (**b**) Diagram of the light path through the Zeiss OPMI Pentero surgical microscope (Carl Zeiss Surgical GmbH); the surgical microscope was equipped with the Blue 400 module which filters the internal broadband xenon light source to excite the surgical field in the 400–410 nm wavelength range and displays the resulting emissions in the 620–710 nm wavelength band. The hyperspectral imaging system (HIS) was attached to a secondary port on the surgical microscope and a liquid crystal tunable filter (LCTF) filtered received light (600–720 nm) generated from the same (Blue 400) excitation.

Spectroscopic algorithms have been developed to decouple PpIX emissions from other fluorophores and eliminate the distorting effects of background optical properties, thereby quantifying the fluorescence that is attributable to the PpIX in tissue. These computations have been applied to localized measurements acquired by a fiberoptic probe device^7, 8^, Fig. 1(a), and yield quantitative PpIX data with significantly improved diagnostic accuracy (relative to measures of fluorescence intensity) when compared to gold-standard cytology^9^. Additional studies focused on probe-based measurements in meningiomas^10^ and low-grade gliomas^11^, which further demonstrated the increased sensitivity to PpIX after spectral analysis and the clinically relevant diagnostic potential of quantitative PpIX evaluation. Similar spectral analyses have since been applied to wide-field imaging of PpIX fluorescence^12^, Fig. 1(b), yielding analogous quantitative estimates capable of characterizing its spatial distribution in tissue providing information not available in the same images of raw fluorescence^13^. While the clinical potential of quantitative fluorescence measurements and wide-field imaging of PpIX has been reported in this series of papers^7–13^, the impact of specific processing steps (including a method for evaluating estimates that approach the limits of signal sensitivity) and their relationships to point probe measurement data has not been studied, but is a prerequisite to addressing the ultimate clinical value of quantitative PpIX evaluation.

Towards this end, the present study considers a series of processing methods for hyperspectral imaging data that (I) estimate the PpIX contribution to collected fluorescence emissions, (II) verify the confidence in the estimate, and (III) correct the estimate for distortions from optical properties. The HIS processing methods are validated through comparison to highly sensitive probe measurements in tissue-simulating phantoms containing clinically-relevant optical properties. Bench-top results show that spectrally-processed estimates of PpIX are more accurate and more sensitive than inspection of visual fluorescence images, and provide improved contrast compared with displays of integrated fluorescence intensity. Application of the methods to *in vivo* cases illustrate where spectrally processed PpIX maps reveal concentrations that are not accessible otherwise. These findings highlight how spectral processing is needed to overcome limitations in system sensitivity and optical property based distortions of PpIX fluorescence within the surgical field.

## Results

### Phantom validation

**Figure 2:**
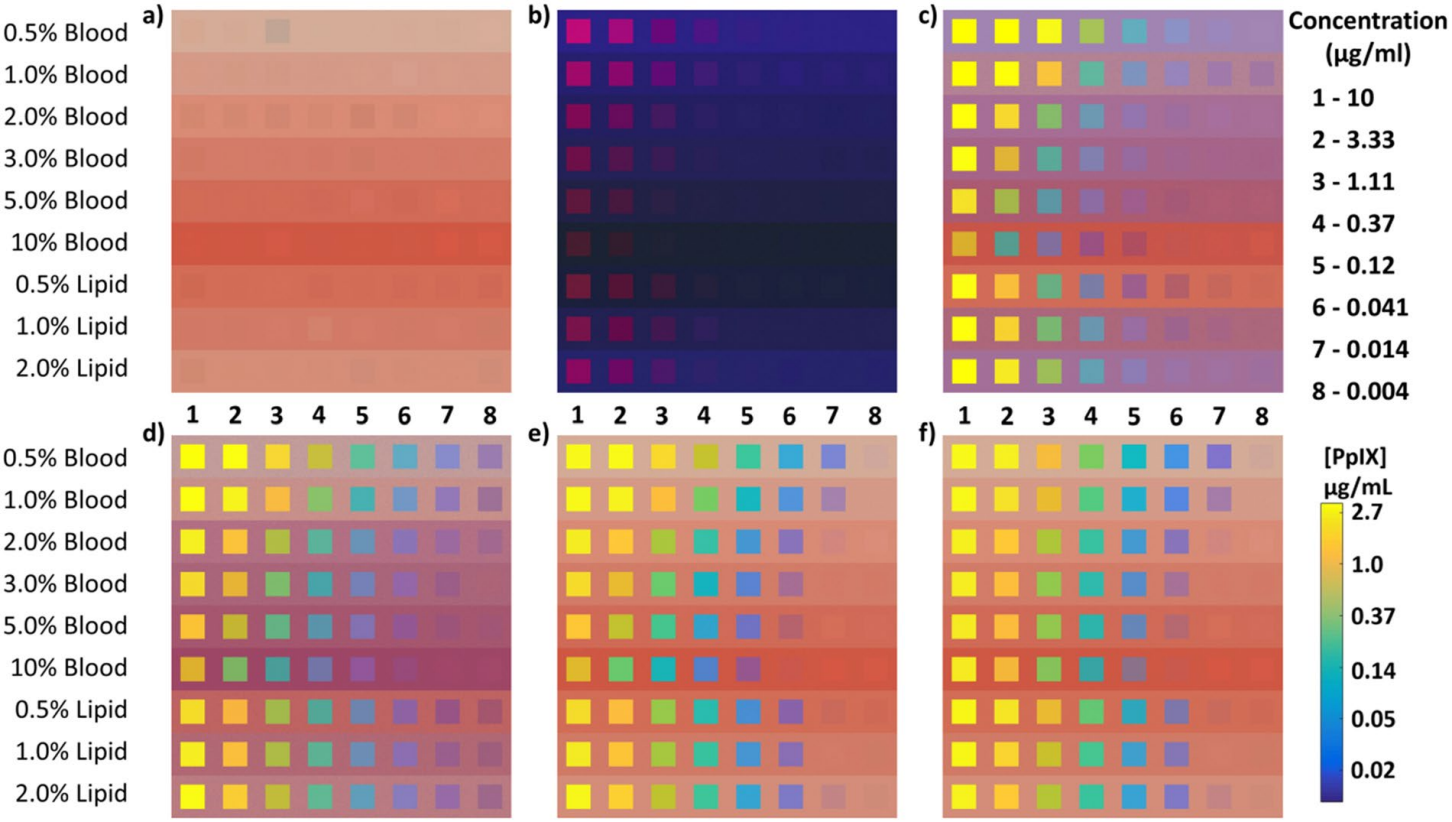
Panels of composite images constructed from experimentally measured phantom data with different processing of PpIX emissions. Within each panel, composite images represent 8 PpIX concentrations (left to right, see upper legend far right) mixed with increasing blood volume fraction (BVF), constant 1.5% lipid volume fraction (LVF), and increasing LVF, constant 2% BVF (top to bottom). The background in each row represents a liquid phantom containing no PpIX. Small variations in the color of each phantom are due primarily to minute differences in blood concentration. (**a**) Reflectance image; pixels were sampled from the white-light images captured by the built-in Zeiss camera. (**b**) Visible fluorescence image; pixels were sampled from Zeiss fluorescence images. (**c**) Integrated fluorescence image; integrated fluorescence intensities ($I{F}_{PpIX}$) were converted to concentration units (see lower legend far right) based on assumed phantom optical properties and overlaid onto the reflectance image in (**a**) using a transparency based on integrated intensity. (**d**) Spectrally fit image; concentration estimates (*SF*_*PpIX*_) based on the same assumed phantom optical properties were overlaid onto the reflectance image in (**a**) using a transparency based on concentration. (**e**) Confidence ratio filtered image; concentration estimates (*SF*_*PpIX*_) were overlaid onto the reflectance image in (**a**) using a transparency based on the confidence ratio (CR). (**f**) Optical property corrected image; concentration estimates (*C*_*PpIX*_) based on optical properties specific to each individual phantom were overlaid onto the reflectance image in (**a**) using a transparency based on the CR.

Figure 2 shows a series of composite images constructed from experimentally measured tissue-simulating phantoms that span a wide range of PpIX concentrations (0.004–10 μg/ml) and optical properties (blood volume fraction (BVF) = [0.5–10]%, lipid volume fraction (LVF) = [0.5–2]%). Each panel within the figure presents a different method for evaluating PpIX emissions, and the series highlights how PpIX quantification is influenced not only by PpIX concentration, but also by attenuation caused by tissue optical properties. Figure 2(a,b) show the white-light and fluorescence appearance of each phantom as a composite of images recorded by the Zeiss camera. Visual estimation of PpIX quantity is influenced significantly by optical properties and is only sensitive to PpIX concentrations between 0.37–1.11 μg/mL depending on their values. Figure 2(c) shows integration of the spectrally-resolved PpIX waveband, *IF*_*PpIX*_, overlaid on the white-light compilation image (converted to concentration units based on assumed phantom optical properties of 2% BVF, 1.5% LVF). Sensitivity and contrast are increased compared to the red channel of the Zeiss image because of a lower noise camera and a narrower wavelength detection band (620–650 nm), respectively. Figure 2(d) displays spectrally fit estimates of PpIX, *SF*_*PpIX*_, obtained from linear spectral decomposition of image-acquired fluorescence spectra and overlaid on the white-light compilation image (also converted to concentration units using the same optical property assumptions). Spectrally fitting the data increases PpIX detection sensitivity and resolves PpIX in additional phantoms within the composite image. The error in the spectrally fit estimates was used to determine the PpIX Confidence Ratio (CR), Equation 6, or the confidence that the fitted spectrum contains contribution from PpIX emissions. These CR values were used as the overlay transparency function (*α*) to filter data based on model confidence, in Fig. 2(e). The CR filter influences the display of PpIX images near the limits of detection. Poorly-informed estimates of PpIX which had a 99.99% confidence interval spanning zero, and therefore were not different from zero (producing *CR* ≤ 0), were visually excluded once overlaid (i.e. *CR* = *α* = 0). As PpIX concentrations decrease from left to right in each row, the CR-filtered image provides clarity about which phantoms containing PpIX are distinguishable from the background. Figure 2(f) shows the previous image corrected for optical properties specific to each phantom in the series, increasing the consistency in the PpIX quantification for changing optical properties (i.e. the color consistency in each column of PpIX concentrations in the composite of images).

**Figure 3:**
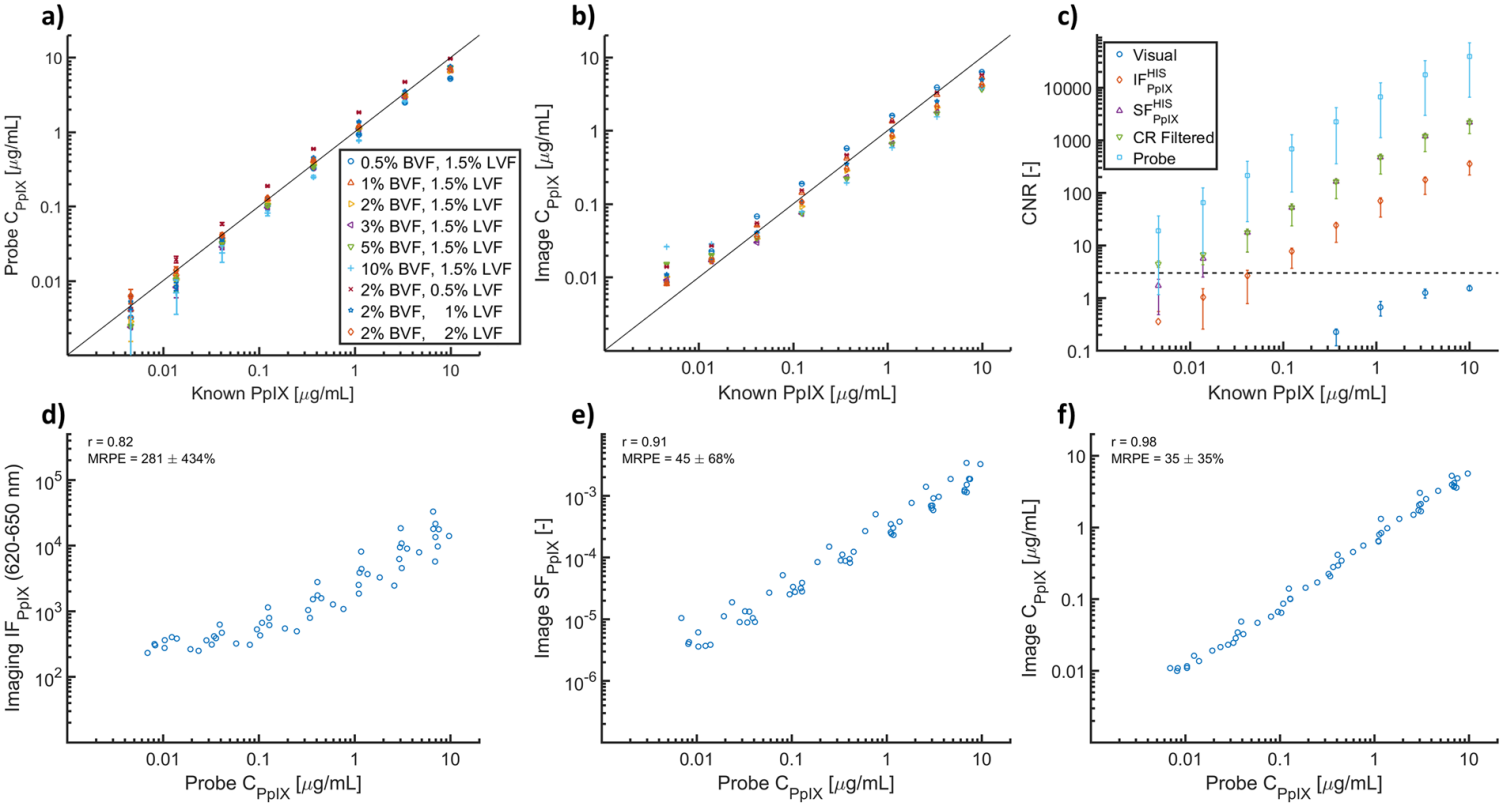
PpIX concentration (*C*_*PpIX*_) estimates from (**a**) spectroscopic probe and (**b**) HIS over a wide range of PpIX concentrations (0.004–10 μg/ml) and optical properties (BVF = [0.5–10]%, LVF = [0.5–2]%), black lines represent unity. (**c**) Contrast-to-noise ratio (CNR) for each system shown as a mean and interquartile range at each PpIX concentration, dotted line represents an CNR of 3 (threshold for lower limit of detection, LLD). Probe *C*_*PpIX*_ compared to HIS estimates of (**d**) integrated fluorescence (*IF*_*PpIX*_), (**e**) spectrally fit fluorescence (*SF*_*PpIX*_), and (**f**) optical property corrected fluorescence (*C*_*PpIX*_).

PpIX sensitivity curves for the spectroscopic probe and HIS are shown in Fig. 3(a,b) for the same set of optical phantoms presented in Fig. 2. A linear relationship exists between estimated and known PpIX for both systems (black lines represent unity). Figure 3(c) shows the contrast-to-noise ratio (CNR) that is achieved by several spectral processing methods vs. PpIX concentration. Here, detectability is defined as a CNR > 3, which is represented by a dashed horizontal line. CNR data displayed in Fig. 3(c) are averaged across all phantom optical properties, while values in Table 1 correspond to HIS-based PpIX estimates for phantoms containing 2% BVF and 1.5% LVF, which is representative of the optical properties of normal cortex. Inspection of the CNR data reveals the effect that spectral processing can have on the lower limit of detection (LLD) for PpIX. Visual fluorescence, taken from Zeiss captures, fails to reach a CNR of 3 for any of the PpIX concentrations tested, highlighting the need for spectral-based processing methods. Integration of the spectrally-resolved waveband improves contrast, and achieves an LLD of 0.12 μg/mL. Spectral fitting further improves the contrast, decreasing the LLD by an order of magnitude to 0.014 μg/mL for most of the optical properties considered. Application of the confidence-based filter results in CNR > 3 over the entire range of PpIX concentrations evaluated, including 0.004 μg/mL; although, at this limit of detection the CR filter only provides PpIX estimates in a fraction of the pixels contained in the phantom image, Table 1. Application of optical-property corrections specific to each individual phantom did not affect the CNR, as shown in Table 1 and are not included in Fig. 2(e). CNR for the probe is higher than for all HIS methods; generating an LLD close to 0.004 μg/mL due in part to a more accurately described background signal, and justifies the use of the probe as a gold-standard in the present study. Figure 3(d–f) compares probe-based estimates of *C*_*PpIX*_ to various metrics of fluorescence from the HIS, including integrated fluorescence (*IF*_*PpIX*_), spectrally fit fluorescence (*SF*_*PpIX*_), and optical property corrected fluorescence (*C*_*PpIX*_), respectively. The data in Fig. 3(d–f) were filtered to exclude PpIX concentrations below 0.014 μg/ml (HIS LLD) and a BVF less than 1%, to eliminate issues with fluorescence correction factors at low absorption. These results clearly demonstrate that accounting for optical property distortions specific to each individual phantom yields a highly linear relationship between probe and HIS *C*_*PpIX*_ estimates (r = 0.98) with reduced variation in the data (mRPE = 35 ± 35%) relative to integrated fluorescence intensity (r = 0.82; mRPE = 281%), or spectral fitting without phantom-specific optical property correction (r = 0.91; mRPE = 45%).

**Table 1:** Contrast-to-noise ratio (CNR) of HIS-based PpIX concentrations estimated in tissue-simulating phantoms representative of normal cortex (2% BVF, 1.5% LVF).

Concentration [μg/mL]	10	3.33	1.11	0.37	0.12	0.041	0.014	0.004
**Integrated Fluorescence**								
Average CNR	344	166	64.8	21.8	6.58	1.83	1.22	0.51
**Spectrally Fit**								
Average CNR	1689	891	344	117	35.0	11.3	4.03	1.46
**CR Filtered**								
Number of pixels	40000	40000	40000	40000	40000	40000	22812	276
Average CNR	1689	891	344	117	35.0	11.3	4.57	3.83
**Corrected Fluorescence**								
Number of pixels	40000	40000	40000	40000	40000	40000	22812	276
Average CNR	1708	878	338	118	36.0	11.3	4.58	3.63

CR-filtered estimates show the number of pixels in the composite phantom image that returned a PpIX estimate with a confidence that was greater than zero.

PpIX estimates in Fig. 2 indicate that the detection limits of PpIX are influenced by optical properties, particularly changes in BVF. The LLD obtained by HIS triples when transitioning from 1% to 2% BVF (0.004 μg/ml to 0.014 μg/ml) and again when transitioning to 3% BVF (0.041 μg/mL) and to 10% BVF (0.12 μg/mL). CR-based filtering has the advantage of not relying on an absolute *C*_*PpIX*_ threshold determined through extensive characterization of the imaging system over a large parameter space. By incorporating the CR-filtering step into the processing, the LLD for PpIX quantification is determined automatically from the strength of the PpIX spectral signature in the collected emission spectrum.

### Patient case illustrations

**Figure 4:**
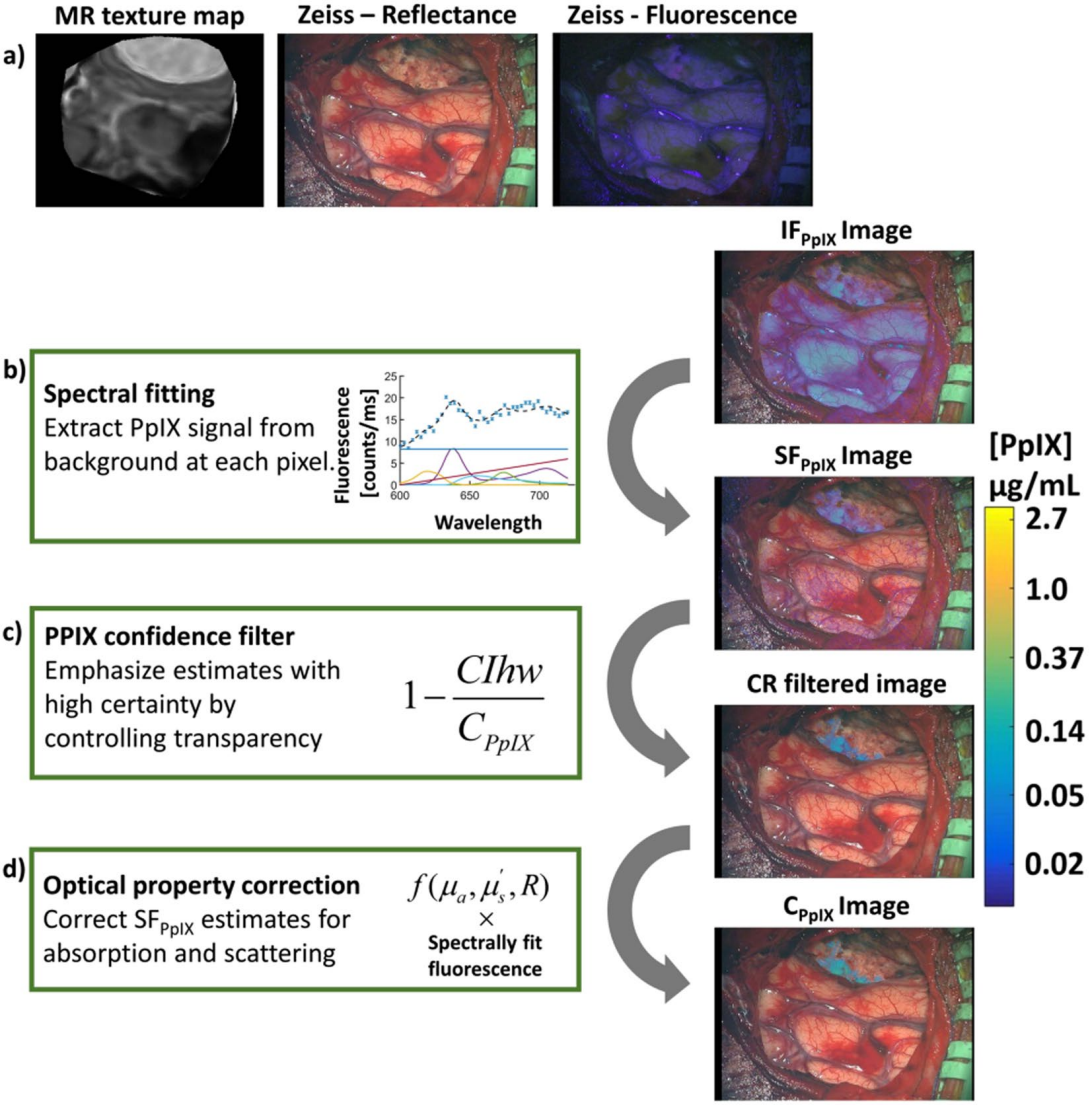
Increased sensitivity to PpIX using wide-field spectroscopic processing is illustrated for a representative case of an atypical meningioma (WHO grade II) towards the end of resection. (**a**) MR-encoded texture map interpolation of the intraoperative location given by the focal point of the operating microscope and the corresponding views of the surgical field under white- and blue-light illumination acquired with the Zeiss Pentero microscope. Log-compressed integrated fluorescence, *IF*_*PpIX*_, is overlaid onto the white-light Zeiss image shown in (**a**), and is analogous to fluorescence observed during FGS. In (**b**) through (**d**), the wide-field spectroscopic techniques are summarized and a simple visual description presented. (**b**) The spectral signature of PpIX is used to extract its contribution from contaminating background signals to produce a spectrally fit PpIX image, *SF*_*PpIX*_. Further processing includes application of (**c**) a transparency function based on the PpIX confidence ratio to the *SF*_*PpIX*_ image to filter noise and poor PpIX estimates and (**d**) an optical property correction specific to the tissues in the surgical field to yield a *C*_*PpIX*_ map. Resulting maps show the specific PpIX biodistribution, which is masked by background fluorescence and specular reflections when using only raw integrated fluorescence.

**Figure 5:**
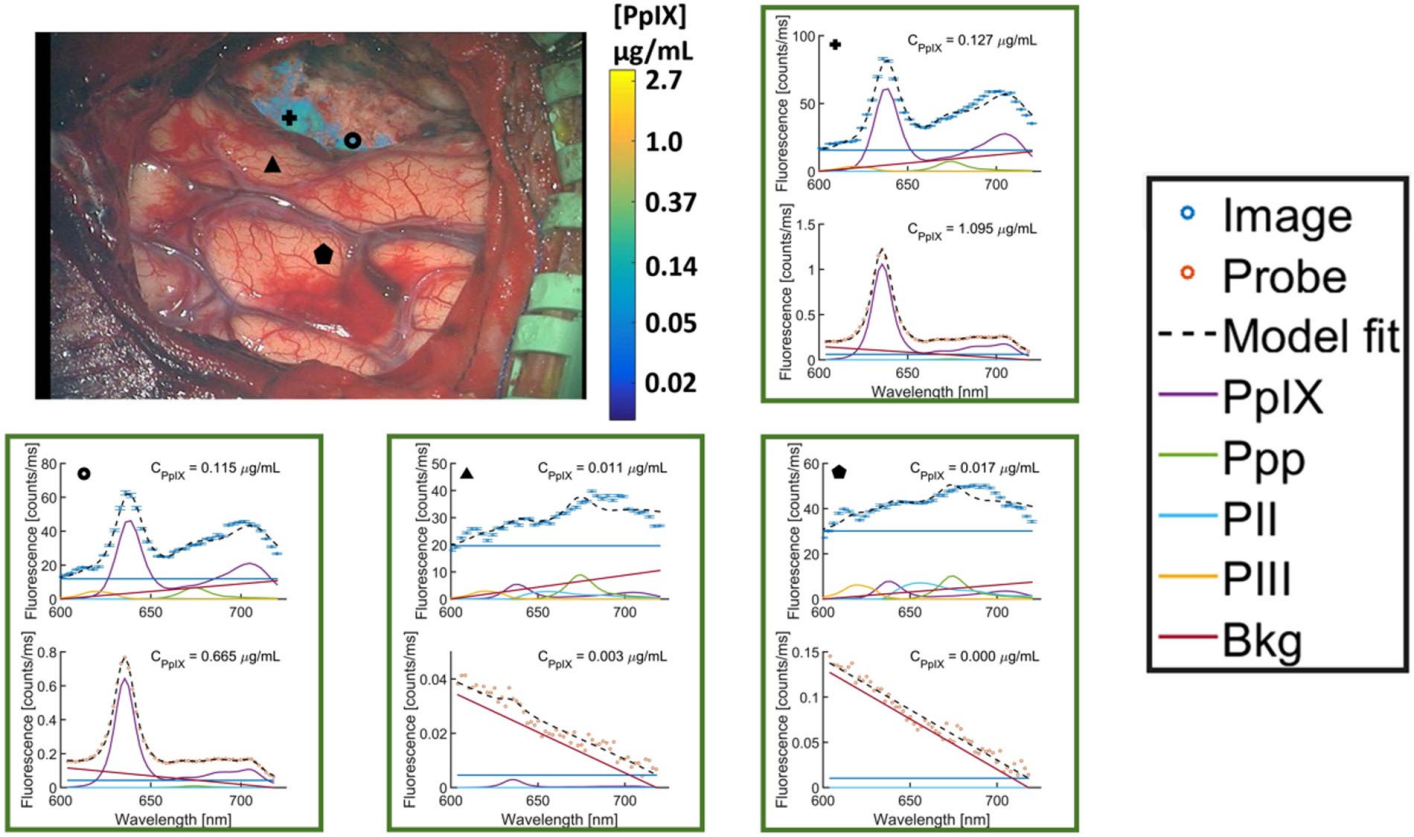
Image (top plot) and probe (bottom plot) spectra at locations corresponding to the symbols shown in the white-light Zeiss image with CR filtered *C*_*PpIX*_ overlay (same as *C*_*PpIX*_ image in Fig. 4). Probe spectra confirm that regions highlighted by the *C*_*PpIX*_ overlay (+ and ○) contain PpIX. Probe measurements in transparent regions of the *C*_*PpIX*_ overlay (Δ and ⬠) confirm the absence of PpIX signal. Ppp = Photoproduct I; PII = Photoproduct II, PIII = Photoproduct III, Bkg = Background, Offset = linear offset for background signal.

Figure 4 illustrates the effect of spectral image processing on identifying PpIX fluorescence in a representative surgical field (atypical meningioma [WHO grade II] towards the end of resection). Figure 4(a) includes an magnetic resonance (MR) texture map of the surgical field interpolated from the coregistered preoperative MR image volume and focal point of the operating microscope tracked in coordinates provided by the navigation system. Corresponding white- and blue-light (fluorescence mode) views of the surgical field observed through the operating microscope are also shown. For comparison, an *IF*_*PpIX*_ image was produced by acquiring a multispectral data set, integrating the raw fluorescence spectra, and overlaying the result on a white-light view of the same surgical field. The *IF*_*PpIX*_ image is similar to the Zeiss fluorescence image in Fig. 4(a) since it is also based on raw fluorescence, and thus serves as a baseline for comparing the spectral processing steps outlined in Fig. 4(b–d). First, the spectral signature of PpIX was used to extract its contribution from contaminating background signal, and generate the *SF*_*PpIX*_ image in Fig. 4(b). One consequence of this procedure was suppression of false positives caused by reflections. Errors in the spectral fits determine the PpIX CR, Equation 6, and were applied to *α* to filter data based on model confidence, Fig. 4(c). In this example, the confidence filter eliminated signals outside the exposed brain, and outside the tumor region not likely arising from PpIX. Thus, the PpIX CR avoids the need to define a concentration threshold by censoring areas with low confidence in the PpIX estimates. Additional processing to correct for tissue-specific optical property distortions in the measured fluorescence yielded *C*_*PpIX*_ maps, Fig. 4(d). Finally, Fig. 5 confirmed that the results generated by these image-processing procedures agreed with probe measurements acquired in the same surgical field. In this comparison, the location of the probe measurements was determined by co-registering HIS images with Zeiss image captures acquired during probe recordings.

**Figure 6:**
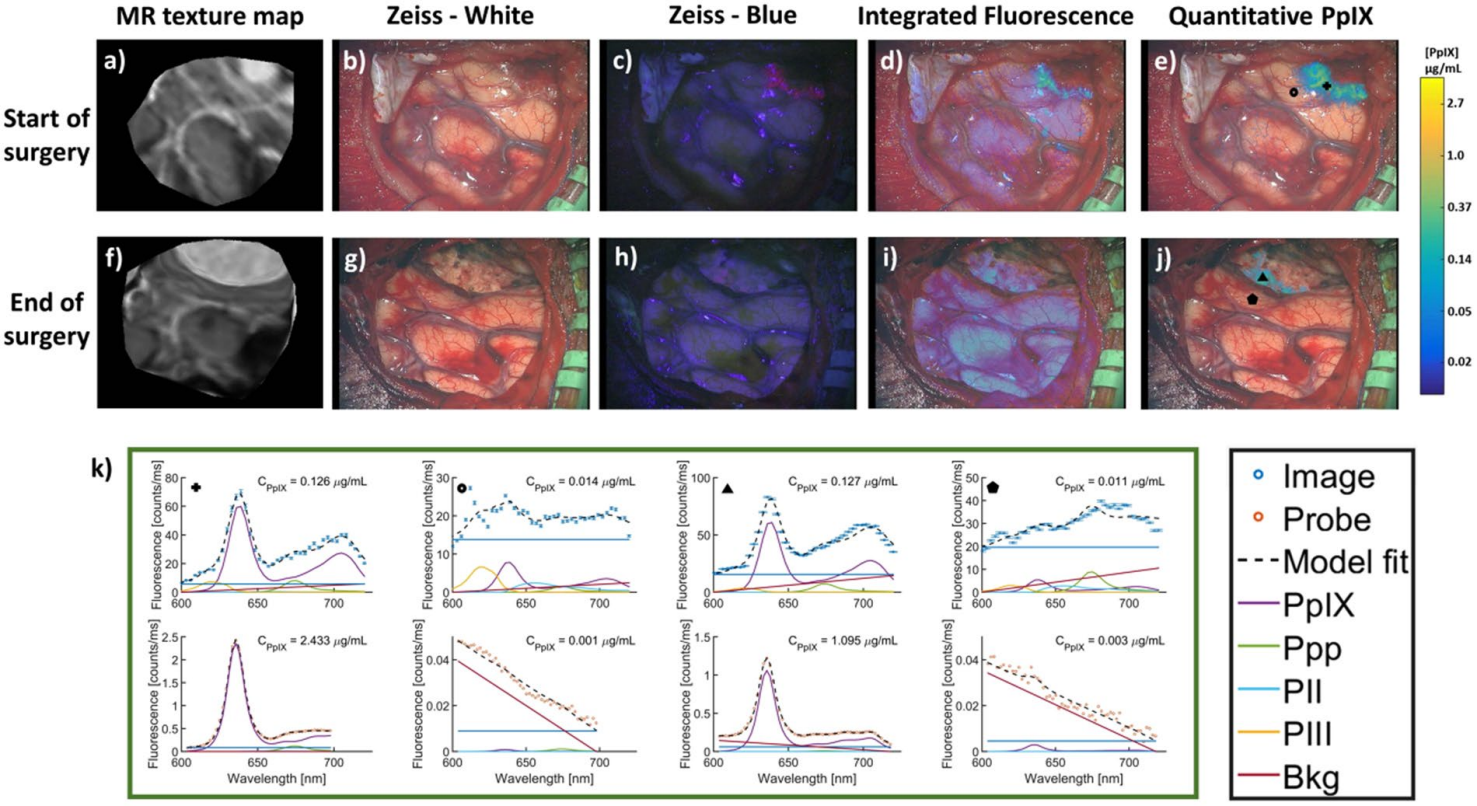
MR-encoded texture map and standard white- and blue-light views of the surgical field taken through the Zeiss Pentero with log-compressed *IF*_*PpIX*_ or PpIX CR filtered *C*_*PpIX*_ overlays at the start (**a**–**e**) and near the end of resection (**f**–**j**) in a patient undergoing surgery for atypical meningioma (WHO grade II). *IF*_*PpIX*_ maps (**d**,**i**) are not well localized at either time point whereas spectrally-processed *C*_*PpIX*_ overlays (**e**,**j**) indicate focal regions of detectable PpIX concentration even towards the end of resection when visually apparent fluorescence has decreased substantially. Comparisons of PpIX spectra (**k**) from image (top plot) and probe (bottom plot) data at the same locations at the start and end of resection [marked by corresponding symbols in the image overlays in (**e**,**j**)] confirm the presence of high and very low (or no) PpIX concentration observed in the *C*_*PpIX*_ overlays. Ppp = Photoproduct I; PII = Photoproduct II, PIII = Photoproduct III, Bkg = Background, Offset = linear offset for background signal.

Images and point spectra from the start and nearing the end of resection for the patient in Fig. 4 are contrasted in Fig. 6 to illustrate the value of *C*_*PpIX*_ images filtered by PpIX CR in terms of localization and contrast relative to visual fluorescence (*IF*_*PpIX*_) over the course of surgery. At the start of resection, mild fluorescence is reported and easily visible to the surgeon, with strong PpIX signal confirmed with both image and probe spectra. The discrepancy in reported quantity between the two systems at marker (+) is likely due to blood accumulation in the field during image-stack acquisition leading to reduced image signal; however, PpIX CR is not influenced by changes in signal magnitude and still confidently detects PpIX. Importantly, towards the end of resection faint fluorescence was reported by the surgeon, which is largely masked by a high background signal, likely cortical autofluorescence, in the *IF*_*PpIX*_ image but readily identified in the *C*_*PpIX*_ map, triangle mark in Fig. 6(j), and confirmed by the probe spectra.

**Figure 7:**
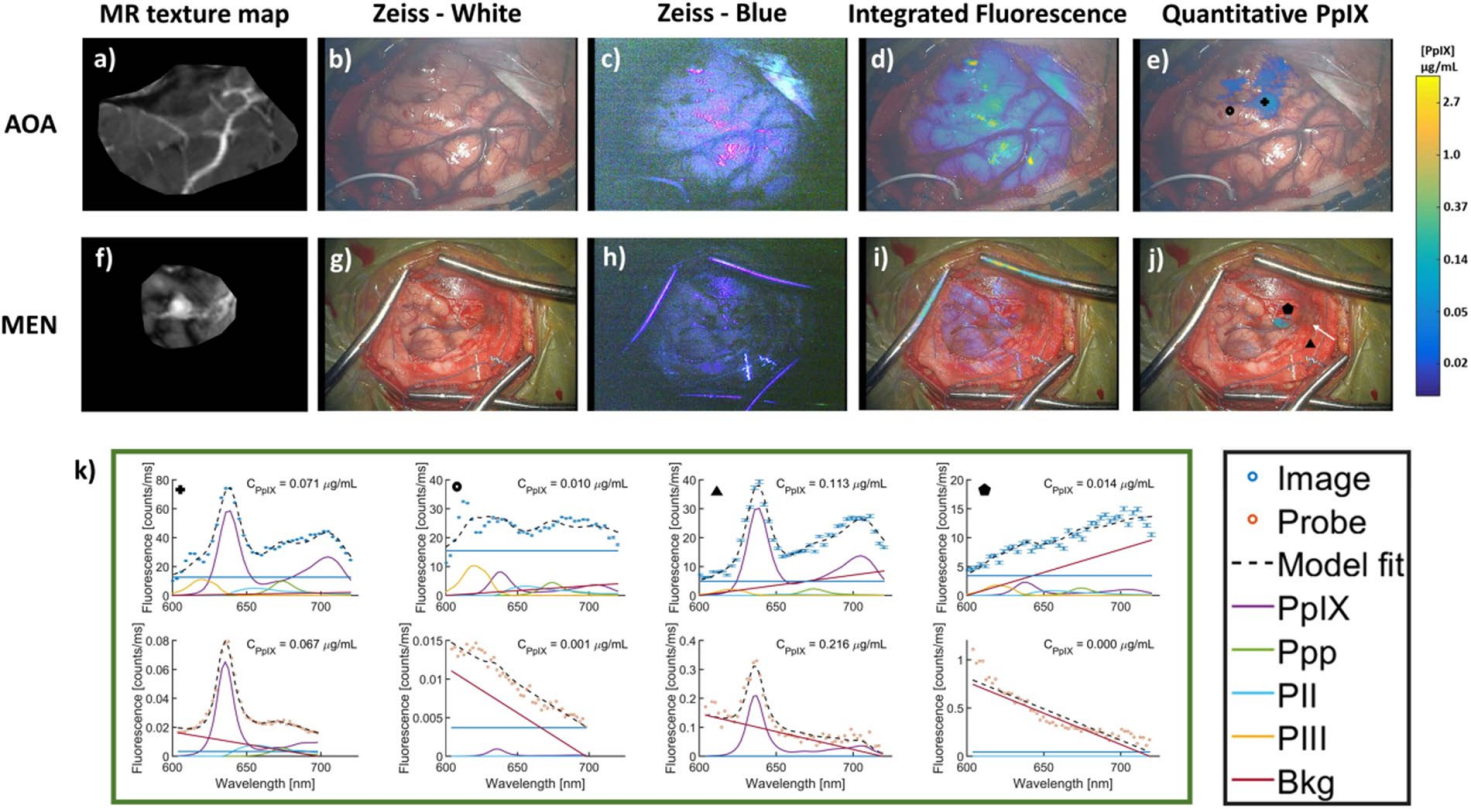
MR-encoded texture map and standard white- and blue-light views of the surgical field through the Zeiss Pentero with log-compressed *IF*_*PpIX*_ or PpIX CR filtered *C*_*PpIX*_ overlays for a patient with an anaplastic oligoastrocytoma (WHO Grade III) (**a**–**e**) and for a patient with a meningioma (WHO grade I) (**f**–**j**). In the first case (top row), the MR-encoded texture map shows no contrast enhancement and the surgeon reported no visible fluorescence as observed with *IF*_*PpIX*_ (**d**), where bright regions correspond to reflections off of the cortical surface, but PpIX is localized in the *C*_*PpIX*_ overlay (**e**). In the second case (bottom row), there is MR contrast enhancement near the image center and faint fluorescence is reported. PpIX fluorescence is not well localized in the *IF*_*PpIX*_ image (**i**) but clearly marked in the *C*_*PpIX*_ image (**j**). Comparisons of PpIX spectra (**k**) from image (top plot) and probe (bottom plot) data at the same locations in the two cases [marked by corresponding symbols in the image overlays in (**e**,**j**)] confirm the presence or absence of PpIX observed in the *C*_*PpIX*_ overlay. Ppp = Photoproduct I; PII = Photoproduct II, PIII = Photoproduct III, Bkg = Background, Offset = linear offset for background signal.

Figure 7 shows images and point spectra for a patient with an anaplastic oligoastrocytoma (WHO Grade III) and for a patient with a meningioma (WHO grade I). In the first case, no MR contrast or visual fluorescence was reported and contamination due to reflections off the cortical surface can be seen in Zeiss blue-light capture and *IF*_*PpIX*_ image, Fig. 7(c,d). Although PpIX fluorescence in tissue shows up as pink/red, reflected excitation light can also appear in the fluorescence imaging channel, as illustrated by the bright pink regions and dura in Fig. 7(c) which correspond to bright white regions (reflections) in Fig. 7(b). This phenomenon can confound the imaging field but spectral processing can filter out contaminated areas, only highlighting areas attributable to PpIX. In the second case, MR contrast and faint visual fluorescence was reported in areas of the surgical field, which are not easily visualized in the Zeiss blue-light capture or *IF*_*PpIX*_ image, Fig. 7(h,i). PpIX CR filtered *C*_*PpIX*_ images, Fig. 7(e,j), localize areas of PpIX that were corroborated with corresponding probe measurements.

## Discussion

The incorporation of wide-field quantification techniques into FGS serves to improve quantitative feedback to the surgeon by providing a map of PpIX across the surgical field. This contrasts the purpose of the probe system, which is to sample a localized (point) tissue volume that the surgeon has deemed of interest either from preoperative imaging or from observations made within the cavity during surgery. While the probe system has proven to accurately quantify PpIX at selected point locations, it is impractical to use the probe to quickly map the distribution of PpIX over large areas of tissue. Imaging allows for mapping the spatial distribution of PpIX with a resolution that the probe system could not provide intraoperatively.

The results of the present study show that the choice of spectral processing steps used to analyze HIS-based fluorescence measurements can have a profound influence on the resulting PpIX map. Results from tissue-simulating phantoms showed that spectral fitting decouples the contribution of PpIX from background signal, that the evaluation of the confidence of fitted PpIX estimates removed poorly-informed estimates of PpIX near the system detection limit, and a diffuse-reflectance based optical property correction was able to compensate for the distortive effects of blood and scattering. Application of the spectral processing steps to *in vivo* case examples during clinical neurosurgeries revealed changes to the localization and contrast of PpIX maps; changes that were likely more representative of the underlying biodistribution of PpIX. Of interest to clinical practice, the data showed that visual fluorescence provided very low contrast-to-noise ratios, which demonstrates the small window of PpIX concentrations that are easily distinguishable from the background and motivates the incorporation of spectral-based analysis into neurosurgical FGS. Notably, a properly trained neurosurgeon may selectively focus on suspicious areas of interest and use experience and contextual information from the surgical field to interpret PpIX emissions. However, this approach is limited to fluorescence that the surgeon can visually perceive, down to 0.37 μg/mL under ideal conditions, based on phantoms with less than 2% BVF, and is qualitative, subjective, and relies heavily on surgeon experience. Comparatively, spectrally-processed data has reliable detectability that is an order of magnitude lower, in the range of 0.014 μg/mL to 0.041 μg/mL, and offers quantitative and objective measures of PpIX fluorescence. The limiting factor in detecting PpIX using spectral fitting is signal; detection of even lower concentrations is possible if a longer exposure or spatial filtering is used to reduce noise, as the contrast of CR filtered pixels remains elevated (CNR > 3) even at the lowest limit of detectable concentrations.

While previous publications have described the methods for spectral fitting and optical property correction of HIS measurements of PpIX^12, 13^, the present study incorporates a novel confidence-ratio technique to filter the wide-field maps automatically and without imposing a specific concentration level threshold. In short, the CR filter removes regions of uncertainty from PpIX maps by excluding poorly-informed estimates that may occur as the system approaches the PpIX detection limits. The filter uses the error in spectral fits (on a pixel-by-pixel basis) to determine the lower limit of detectability rather than relying on absolute PpIX concentration cutoffs to define bounds on signal sensitivity. Setting a fixed lower limit *a priori* for the displayed concentration is confounding when imaging through an operating microscope because the lower bound of detection varies with lamp intensity, focal length, zoom, and external light sources in addition to tissue optical properties. In a continually shifting operating room environment, a fixed concentration limit may result in detectable PpIX being removed or poorly-informed PpIX estimates being displayed. In Fig. 2 the transition from panel (d) to (e) showcases CR filter advantages, yielding a drastic reduction in background signal and removal of PpIX estimates from some low concentration phantoms. These results suggest that the CR filter has the potential to reduce false-positives, represented by the background in Fig. 2, by removing data with high PpIX uncertainty.

The application of the spectral processing methods to clinical neurosurgical FGS data, as shown in Figs 4, 5, 6 and 7, illustrates surgical examples where *C*_*PpIX*_ images returned by spectral processing offer improved localization and contrast relative to current clinical standards and integrated intensity maps. Locations of potential surgical significance were highlighted, and the more accurate representation of the biodistribution of PpIX presented in the *C*_*PpIX*_ image was confirmed with independent optical probe measurements of PpIX concentration. While strong agreement is observed in the comparisons noted throughout the clinical case examples, discrepancies in the absolute value of *C*_*PpIX*_ reported by the probe and HIS can occur for multiple reasons. First, the two optical measurement systems sample different tissue volumes. The HIS samples fluorescence emissions at each pixel location that are multiply scattered and therefore blurred/mixed with contributions from surrounding locations in the tissue. Conversely, the fiber optics of the probe geometrically localize the fluorescence emissions within a small volume of tissue located in close proximity to the probe tip. Second, the HIS samples a tissue surface that is not flat and will include height- and orientation-based variations in collected fluorescence intensity, while the probe is placed in gentle contact with the tissue surface and (as long as good contact is achieved) is unaffected by topographic variations. Third, the HIS assembles spectra at each pixel in the imaged field by collecting sequential images at different wavelengths, and even though each image is quickly acquired (50 ms for reflectance, 250 ms for fluorescence) movement (e.g. from breathing, etc.) is expected introduce some blurring between the origin and coregistered location. Fourth, the longer acquisition time of the HIS compared with the probe is susceptible to temporal changes occurring in the surgical field during the measurement, including blood accumulation within the surgical cavity during acquisition of the HIS image stack, which can mask PpIX fluorescence. The latter effect can be mitigated by using red-light (635 nm) excitation of PpIX^14^. These discrepancies further advocate for a concentration-independent threshold filter and demonstrate the value of the CR filter for localizing PpIX.

Availability of accurate and quantitative *C*_*PpIX*_ images have the potential to catalyze application of ALA mediated FGS to other brain tumor histologies. Low-grade gliomas are of particular interest; studies have reported a lack of visual fluorescence^1, 15, 16^ even though PpIX has been detected with microscopic^17^ and spectroscopic^9, 11, 18, 19^ techniques. While the presence of PpIX-based contrast in low-grade gliomas has been confirmed, the low concentrations of PpIX make visualization with current FGS methods more difficult. The processing techniques evaluated here are ultimately limited by the sensitivity of the current HIS to PpIX, but can be easily applied to more sensitive systems as they become available.

The study described in this paper focused on the importance of spectral processing of the raw fluorescence signals for yielding accurate maps of the PpIX biodistribution; the data reported do not build the link between the detected PpIX concentration and the underlying tissue pathology. Determination of diagnostic performance requires more substantial cataloging of clinical data, including detailed comparisons with histology for definitive confirmation. Previous work has examined the diagnostic performance of our probe system for various tumor histologies^8–11^ and the results in this study provide the tools necessary to conduct analogous investigations with our HIS in the future. Additionally, while this study focused on the detection of PpIX, these techniques can be applied to other HISs and/or imaging windows for the detection of other fluorescent markers. For example, near-infared (NIR) imaging penetrates deeper into tissue due to significantly reduced absorption of blood and other tissue fluorophores, low scattering, and minimal tissue autofluorescence^20^, but requires augmented display since NIR light is not directly visible to the human eye.

## Methods

### Phantom preparation

Liquid phantoms were constructed, as described previously^21^, to characterize the PpIX fluorescence spectral response sampled over a range of background optical properties and fluorophore concentrations. The phantom set incorporated variations in blood volume fraction (BVF) of (0.5, 1, 2, 3, 5, 10)% with a constant lipid volume fraction (LVF) of 1.5% and PpIX concentrations in the range of (0.004–10) μg/ml, in three-fold dilutions. Additionally, phantoms with a fixed BVF of 2% were made with variations in LVF of (0.5, 1, 2)% for a total of 81 combinations, including baseline phantoms with no PpIX for each combination. Optical data were recorded with both the probe and HIS, and consisted of sequences of reflectance measurements acquired under white-light illumination and fluorescence measurements obtained under blue-light illumination.

Image processing steps were validated with composite images generated from this phantom set. Phantom composite images were made by randomly selecting a sample of 40,000 pixels from each phantom to form a 200 × 200 pixel square representing each liquid phantom containing PpIX, generating a 9 × 8 grid. Each phantom contained roughly 45,000 usable pixels after removing those saturated by specular reflections or containing bubbles. The background for each row was generated by randomly selecting 980,000 pixels from a liquid phantom containing no PpIX to form a 2800 × 350 pixel rectangle; resampling filled the background row. These compilation images provided flexibility and control in the design of a phantom that explored a wide range of PpIX concentrations and background optical property variations, while avoiding boundary effects associated with heterogeneous phantoms containing inclusions. The compilation images provides insight into the effect of spectral processing on pixel-by-pixel basis, but provide no information about spatial localization or resolution limits of heterogeneities.

### Spectroscopic equipment

Optical measurements were acquired with a custom-built fiberoptic probe and hyperspectral imaging system (HIS), both described in detail previously^7, 12^. Briefly, the hand-held probe contained four active fiber optic leads of 200 μm in diameter: a detector fiber [connected to a USB2000+ spectrophotometer (Ocean Optics, Dunedin, FL)], a blue LED (405 nm) source (LedEngin Inc. Santa Clara, California) fiber, and two fibers that lead to a white LED light source (LedEngin Inc. Santa Clara, California), Fig. 1(a). A typical measurement sequence consisted of a blue-light measurement, two white-light measurements, and a dark measurement taking about 4 seconds to complete. Each measurement uses test exposures to automatically adjust exposure time and averages signal over repeated measurements within a 500 ms acquisition period. The HIS used in this study was an updated version an earlier device^12^. It consisted of three main components: a custom optical adapter, a liquid crystal tunable filter (LCTF) (Cambridge Research Instruments), and a complementary metal-oxide semiconductor (CMOS) camera (PCO.Edge, Cooke Corp.) connected to a computer control system. The custom adapter attached the HIS to a side-port of the Zeiss OPMI Pentero surgical microscope (Carl Zeiss Surgical GmbH), and spectral response data was acquired through the microscope light path, Fig. 1(b). Excitation light was provided by the Zeiss Blue 400 module functioning in its standard mode of clinical operation, and was not otherwise altered or adjusted during use in this study (blue-light intensity from the unit was measured to be 7 mW/cm^2^). The HIS recorded a fixed number of wavelengths at a fixed integration time of 50 ms for reflectance and 250 ms for fluorescence, resulting in reflectance measurements from 440–720 nm (4 nm spacing, 7 seconds) and fluorescence measurements from 600–720 nm (3 nm spacing, 12 seconds). The same fluorescence imaging setup described here was used for both benchtop and clinical measurements.

### Data analysis

#### Processing of Fiberoptic Probe Measurements

Raw white-light reflectance spectra measured by the probe were calibrated by subtracting the background signal, dividing by integration time, and then normalizing by a ratio of the model-estimated and measured reflectance spectra of an Intralipid reference phantom containing 2% LVF. Work by Kim *et al*.^22^ details the probe reflectance analysis model, which expresses diffuse reflectance intensity in terms of the absorption coefficient (*μ*_*a*_), reduced scattering (${\mu }_{s}^{^{\prime} }$), and source-detector separation (*ρ*), such that $R(\lambda )=f({\mu }_{a}(\lambda ),{\mu }_{s}^{^{\prime} }(\lambda ),\rho )$. Tissue ${\mu }_{s}^{^{\prime} }$ was estimated using a wavelength dependent power law^23^,

1 $${\mu }_{s}^{^{\prime} }(\lambda )={\mu }_{s}^{^{\prime} }({\lambda }_{o})\,{(\tfrac{\lambda }{{\lambda }_{o}})}^{-b},$$

where ${\mu }_{s}^{^{\prime} }({\lambda }_{o})$ and *b*, the scattering slope, are fitted values. Tissue *μ*_*a*_ was modeled as a linear sum of significant chromophores,

2 $${\mu }_{a}(\lambda )={C}_{ves}BVF[St{O}_{2}{\varepsilon }_{a}^{oxyHb}(\lambda )+(1-St{O}_{2}){\varepsilon }_{a}^{deoxyHb}(\lambda )],$$

where *BVF* is the blood volume fraction, *StO*_2_ is the microvascular saturation, and ${\varepsilon }_{a}^{oxyHb}$ and ${\varepsilon }_{a}^{deoxyHb}$ are the wavelength-dependent specific absorption coefficients [1/mm] of fully oxygenated and deoxygenated hemoglobin, respectively. *C*_*ves*_ is a correction factor to account for the distortive influences that heterogeneous distributions of blood vessels have on the effective absorption coefficient^24^. The factor is given by ${C}_{ves}=[1-{e}^{(-2{\mu }_{a}^{blood}{r}_{v})}]/[2{\mu }_{a}^{blood}{r}_{v}]$, where ${\mu }_{a}^{blood}$ is the *μ*_*a*_ attributable to blood and *r*_*v*_ is the effective mean vessel radius, which was set to 8 (the size of a red blood cell). Thus, nonlinear spectral fitting, using *lsqnonlin*, of white-light reflectance spectra yielded estimates of *μ*_*a*_ and ${\mu }_{s}^{^{\prime} }$ using four fitted parameters [${\mu }_{s}^{^{\prime} }({\lambda }_{o})$, *b*, *BVF*, and *StO*_2_]. These properties were used extrapolate the reduced albedo at excitation wavelength (${a}_{x}^{^{\prime} }={\mu }_{s,x}^{^{\prime} }/({\mu }_{a,x}+{\mu }_{s,x}^{^{\prime} })$), which can be used to calculate the total diffuse reflectance at excitation (*R*_*t*,*x*_)^25^. Raw fluorescence spectra measured by the probe were calibrated by subtracting the background signal and dividing by integration time. Quantitative fluorescence returned by the probe (${F}_{C}^{probe}$) was determined as previously described^7^ from

3 $${F}_{C}^{probe}(\lambda )=(\frac{{\mu }_{a,x}}{1-{R}_{t,x}})\,(\frac{{F}_{meas}(\lambda )}{{R}_{m}}),$$

where *μ*_*a*,*x*_ is the absorption coefficient at the excitation wavelength of 405 nm, and *R*_*m*_ is the calibrated diffuse reflectance measured at 635 nm; this work is described extensively elsewhere^7, 22^. Spectral fitting of the probe-based quantitative fluorescence spectrum yields corrected estimates of contributions from each fluorophore [${C}_{PpIX}^{probe}$, ${C}_{PP}^{probe}$, and ${C}_{Bkg}^{probe}$], which are still in [a.u.] due to the use of unitless basis spectra (normalized to a peak of 1). The corrected estimates of PpIX (*C*_*PpIX*_) were converted from a.u. to μg/mL by applying a linear scaling factor, ${\beta }_{PpIX}^{probe}$, which was determined by minimizing the error between PpIX estimates and known concentrations in a set of reference phantoms with 2% BVF and 1.5% LVF: ${C}_{PpIX,scaled}^{probe}={\beta }_{Ppix}^{probe}{C}_{PpIX,unscaled}^{probe}$. Processing of probe spectra in custom MATLAB scripts required roughly 1 second to complete.

#### Processing of Hyperspectral Imaging Measurements

HIS images were calibrated by dividing by a measurement of spectralon (99%) to correct for nonuniform light distribution across the field-of-view. Fluorescence spectra sampled by the HIS were analyzed three different ways to yield three different metrics of PpIX: (I) integrated fluorescence (*IF*_*PpIX*_), (II) spectrally fit PpIX (*SF*_*PpIX*_), and (III) corrected or quantitative PpIX (*C*_*PpIX*_). Integrated fluorescence was calculated as the area under the fluorescence spectrum from 620 nm to 650 nm. Spectral fitting isolated characteristic PpIX signatures by linear unmixing; measured spectra were assumed to be a linear combination of emission profiles from PpIX, PpIX photoproducts, and background signal, returning the parameter set [$S{F}_{PpIX}^{HIS}$, $S{F}_{PP}^{HIS}$, and $S{F}_{Bkg}^{HIS}$] which are estimates of the fluorescence contribution [a.u.] of PpIX, PpIX photoproducts, and background, respectively. Here, background is modeled as a linear function of wavelength with positive or negative slope, the former accounting for white light bleeding through the source-side filters in the Zeiss Blue 400 module (which filters the white-light source to create its blue-light exposure when functioning in fluorescence imaging mode), which can be larger than the (negatively sloped) tissue autofluorescence. Quantitative PpIX was calculated using a correction algorithm to remove distortions from optical properties, which included a simple ratio of fluorescence and reflectance bands at excitation and emission. Quantitative fluorescence returned by the HIS (${F}_{C}^{HIS}(\lambda )$) was determined as described previously^12^

4 $${F}_{C}^{HIS}(\lambda )=\frac{{F}_{meas}(\lambda )}{{R}_{x}{({R}_{m})}^{-0.7}},$$

where *F*_*meas*_(*λ*) is the measured fluorescence in [counts/ms], *R*_*x*_ represents measured reflectance at the excitation band (465–485 nm), and *R*_*m*_ is the measured reflectance at the emission band (625–645 nm). An excitation band of 465–485 nm was chosen due to detection side filters in the Pentero microscope, which did not allow for measurement below 450 nm. Raising *R*_*m*_ to a power of −0.7 served to account for scattering attenuation at emission. Spectral fitting of ${F}_{C}^{HIS}$ returns estimates of corrected fluorescence [${C}_{PpIX}^{HIS}$, ${C}_{PP}^{HIS}$, ${C}_{Bkg}^{HIS}$] [a.u.]. Conversion from [a.u.] to [μg/mL] was again done by applying a linear scaling factor, ${\beta }_{PpIX}^{HIS}$, which was determined from measured phantoms. over a wide range of optical properties and PpIX concentrations. Data processing was performed in custom coded MATLAB scripts (2015a, Mathworks, Natick, MA USA), and spectral processing of HIS data required about 4 seconds per image (1.5–2 seconds of which is image registration).

### Statistical analysis

Model analysis of reflectance and fluorescence spectra used the coefficient of determination (*R* ^2^) as the metric for goodness of fit. Model fits that returned *R* ^2^ values below 0.9 for either reflectance or fluorescence were identified as poor fits and excluded from analysis. For the HIS, spectral fitting was performed on a pixel-by-pixel basis but results are presented as the arithmetic mean of pixels in a region of approximately 1 mm in diameter (n = 201 pixels), roughly matching the diameter of the fiberoptic probe tip. Thus, imaging parameter estimates are presented as means and standard errors over approximately 201 pixels. To prevent magnification of noise or suppression of data in pixels with reflectance spectra of poor quality, the fluorescence correction factor in Equation 4 for the HIS was bounded between 0.2 and 10, based on correction factor values observed in tested phantoms. To estimate the lower limit of detection (LLD), contrast-to-noise ratio (CNR) was determined for each system at each PpIX concentration. CNR was chosen as an indication of both the quality of the signal and its contrast, since both are critically important for surgical guidance. CNR was defined as

5 $$CNR=\frac{{\mu }_{target}-{\mu }_{bkg}}{{\sigma }_{bkg}},$$

where *μ*_*target*_ is the average fluorescence metric (*IF*_*PpIX*_, *SF*_*PpIX*_, or *C*_*PpIX*_) over a 40,000 pixel ROI in the phantom of interest, *μ*_*bkg*_ is the average fluorescence metric in a optical property matched phantom with no PpIX, and *σ*_*bkg*_ is the standard deviation of the fluorescence metric in a optical property matched phantom with no PpIX. For visual fluorescence, the intensity of the red channel was normalized by the sum of all three color channels to allow for a rudimentary optical property correction that may better represent how a surgeon experienced with PpIX perceives the concentration. This step also compresses the information to a single value for each pixel that can be used to calculate the CNR.

Following previous work by Amelink *et al*.^26^, confidence intervals (CI) were determined for spectrally fit parameters in our reflectance and fluorescence analysis algorithms. CIs represent the uncertainty of an estimated parameter value, and we used CIs to inform a filter to automatically identify and reject poorly informed estimates of PpIX. The PpIX confidence ratio (CR) was defined as

6 $$CR=1-\frac{C{I}_{hw}}{S{F}_{PpIX}},$$

where *CI*_*hw*_ is the 99.99% confidence interval half-width for the estimated value of PpIX. A stringent CI was chosen to conservatively map the PpIX biodistribution and ensure that overlays were constructed using only highly reliable estimates. Normalizing by the parameter estimate and subtracting from unity returns values over a range of 0 to 1. Negative values (i.e. *CI*_*hw*_ > *SF*_*PpIX*_) signify that the estimated parameter value is not different from zero. For aggregate phantom data, mean relative percentage error (mRPE) was used to quantify differences between PpIX fluorescence metrics that were returned by the probe and HIS. Probe estimates were plotted against HIS estimates and mRPE was determined from the best fit line through the data passing through the origin. Thus, mRPE is defined as the sum of the absolute value of relative errors $(|\tfrac{actual-predicted}{predicted}|)$ divided by the number of values, where the predicted value was determined from the best fit line.

### *In vivo* study characteristics

Data analyzed in this study were collected as part of a broader enrollment of surgical patients with a variety of brain tumor histologies following a protocol that was approved by the Committee for the Protection of Human Subjects at Dartmouth. Informed consent was obtained from participants included in the study. All research procedures involving human participants were approved by the Dartmouth Institutional Review Board. An oral dose of ALA (DUSA Pharmaceuticals) was prepared by dissolving 20 mg/kg in 100 ml of water and was administered approximately 3 hours prior to induction of anesthesia. Preoperative high-resolution, contrast-enhanced T1-weighted or T2-weighted MR images were acquired and used for image-guided neuronavigation during each case.

Patients were positioned in a 3-point pin fixation. A StealthStation image-guidance system (Medtronic, Louisville, CO, USA) provided the neuronavigation following standard practice. Preoperative MR (pMR) data were co-registered with the surgical field using scalp fiducials, and a Zeiss OPMI Pentero surgical microscope (Carl Zeiss Surgical GmbH) equipped with the BLUE 400 fluorescence imaging module was tracked. Resection was carried out following standard microsurgical technique. The surgeon alternated between white and blue light-emitting modes to visualize fluorescence during the case. During spectral image acquisition, the surgical microscope was set to a zoom of 2.6x and a focus of 300 nm. The surgeon then placed the intraoperative fiberoptic probe on several sites of interest in the surgical field, and quantitative PpIX measurements were recorded. During each probe measurement site two Zeiss images were taken as part of the sequence, one image was acquired with the probe lights on to allow exact visualization of the probe and the other with the lights off to visualize fluorescence emissions around the probe tip. The additional images add 2 seconds to the probe sequence. Control data were acquired from normal cortex that did not undergo resection during these procedures. Measured sites were also assigned a qualitative visible fluorescence score from 0 to 3 as described previously^27^ (0, no fluorescence; 1 minimal fluorescence; 2, moderate fluorescence; and 3, high fluorescence). Resection was continued until the surgeon judged that no more tissue could be safely removed.

### Visualization techniques

Magnetic resonance (MR) texture maps were generated as detailed in ref. 28. Briefly, preoperative MR (pMR) images were segmented and intraoperative stereovision (iSV) data were transformed to match the MR coordinate system. The iSV data was then sampled and projected onto the pMR segmentation to geometrically match the pMR brain surface. Finally, the projected iSV intensities were replaced with interpolated values from the corresponding pMR image to generate the MR-encoded texture maps. A transparency function was used to overlay pseudo-colored fluorescence images on white-light views of the surgical field. The transparency was described by *α* specified at each pixel within in an image, with values ranging from 0 to 1 (where 1 is opaque and 0 is completely transparent). Transparency maps for *IF*_*PpIX*_ and *SF*_*PpIX*_ were generated by linearly scaling data within an image to a range of 0 to 1 using fixed upper (UB) and lower (LB) bounds (i.e. *α* = (*data* − *LB*)/(*UB* − *LB*)). Bounds for *IF*_*PpIX*_ and *SF*_*PpIX*_ were $[5432,\,109098]$ and $[0.01,\,4.5]$, respectively. When the PpIX confidence ratio (CR) filter was applied to *SF*_*PpIX*_ or *C*_*PpIX*_, the estimated CR values were directly used as the *α* value. By encoding the confidence estimate into the transparency function, the criterion for display of PpIX within an image was based on the quality of PpIX estimated at each pixel within the image.

Overlays of HIS data were plotted on the same colorbar and scale. The unified color scale better visualized the effect of each processing step on the data. Since *IF*_*PpIX*_ and *SF*_*PpIX*_ are normally in a.u., optical properties were assumed in order to convert the estimates into units of concentration (μg/mL) for direct comparison with *C*_*PpIX*_. The PpIX scaling factor, *β*_*PpIX*_, determined for *C*_*PpIX*_ was applied to *SF*_*PpIX*_ images in order to use the same color scale. Overlay generation required less than a second once the imaging data had been processed.

### Data availability

The datasets generated and/or analyzed during the current study are available from the corresponding author on reasonable request.

## References

[1] Stummer, W. et al. Intraoperative detection of malignant gliomas by 5-aminolevulinic acid-induced porphyrin fluorescence. Neurosurg. **42**, 518–25, discussion 525–6, doi:10.1097/00006123-199803000-00017 (1998). 10.1097/00006123-199803000-00017 9526986

[2] Stummer W (2006). Fluorescence-guided surgery with 5-aminolevulinic acid for resection of malignant glioma:a randomised controlled multicentre phase III trial. Lancet. Oncol..

[3] Collaud S, Juzeniene A, Moan J, Lange N (2004). On the selectivity of 5-aminolevulinic acid-induced protoporphyrin IX formation. Curr. Med. Chem. Anticancer. Agents.

[4] Mansfield JR, Gossage KW, Hoyt CC, Levenson RM (2005). Autofluorescence removal, multiplexing, and automated analysis methods for in-vivo fluorescence imaging. J. Biomed. Opt..

[5] Müller M, Georgakoudi I, Zhang Q, Wu J, Feld M (2001). Intrinsic fluorescence spectroscopy in turbid media: disentangling effects of scattering and absorption. Appl. Opt..

[6] Keijzer M, Richards-Kortum RR, Jacques SL, Feld MS (1989). Fluorescence spectroscopy of turbid media: autofluorescence of the human aorta. Appl. Opt..

[7] Kim A, Khurana M, Moriyama Y, Wilson B (2010). Quantification of in vivo fluorescence decoupled from the effects of tissue optical properties using fiber-optic spectroscopy measurements. J. Biomed. Opt..

[8] Valdés PA (2011). Combined fluorescence and reflectance spectroscopy for in vivo quantification of cancer biomarkers in low- and high-grade glioma surgery. J. Biomed. Opt..

[9] Valdés PA (2011). Quantitative fluorescence in intracranial tumor: implications for ALA-induced PpIX as an intraoperative biomarker. J. Neurosurg..

[10] Valdés, P. A. et al. 5-aminolevulinic acid-induced protoporphyrin IX fluorescence in meningioma: qualitative and quantitative measurements *in vivo*. Neurosurg. 10 Suppl 1, 74–82, discussion 82–3, doi:10.1227/NEU.0000000000000117 (2014). 10.1227/NEU.0000000000000117 PMC4237006 23887194

[11] Valdés PA (2015). Quantitative fluorescence using 5-aminolevulinic acid-induced protoporphyrin IX biomarker as a surgical adjunct in low-grade glioma surgery. J. Neurosurg..

[12] Valdés PA (2012). A spectrally constrained dual-band normalization technique for protoporphyrin IX quantification in fluorescence-guided surgery. Opt. Lett..

[13] Valdés PA (2012). Quantitative, spectrally-resolved intraoperative fluorescence imaging. Sci. Rep..

[14] Roberts, D. W. *et al*. Red light excitation of protoporphyrin IX fluorescence for subsurface tumor detection. J. Neurosurg. doi:10.3171/2017.1.JNS162061 (2017). 10.3171/2017.1.JNS162061 PMC5797501 28777025

[15] Hefti M (2008). 5-aminolaevulinic acid-induced protoporphyrin IX fluorescence in high-grade glioma surgery. Swiss Med. Wkly..

[16] Widhalm G (2010). 5-aminolevulinic acid is a promising marker for detection of anaplastic foci in diffusely infiltrating gliomas with nonsignificant contrast enhancement. Cancer.

[17] Sanai N (2011). Intraoperative confocal microscopy in the visualization of 5-aminolevulinic acid fluorescence in low-grade gliomas: clinical article. J. Neurosurg..

[18] Utsuki S (2006). Possibility of using laser spectroscopy for the intraoperative detection of nonfluorescing brain tumors and the boundaries of brain tumor infiltrates: Technical note. J. Neurosurg..

[19] Ishihara R (2007). Quantitative spectroscopic analysis of 5-aminolevulinic acid-induced protoporphyrin IX fluorescence intensity in diffusely infiltrating astrocytomas. Neurol. medico-chirurgica.

[20] Gioux S, Choi HS, Frangioni JV (2010). Image-guided surgery using invisible near-infrared light: fundamentals of clinical translation. Mol. Imaging.

[21] Marois M, Bravo J, Davis SC, Kanick SC (2016). Characterization and standardization of tissue-simulating protoporphyrin ix optical phantoms. J. Biomed. Opt..

[22] Kim, A., Roy, M., Dadani, F. & Wilson, B. A fiberoptic reflectance probe with multiple source-collector separations to increase the dynamic range of derived tissue optical absorption and scattering coefficients. *Opt. Express* 18, doi:10.1364/OE.18.005580 (2010). 10.1364/OE.18.005580 20389574

[23] Doornbos RM, Lang R, Aalders MC, Cross FW, Sterenborg HJ (1999). The determination of *in vivo* human tissue optical properties and absolute chromophore concentrations using spatially resolved steady-state diffuse reflectance spectroscopy. Phys. Med. Biol..

[24] Rajaram N, Gopal A, Zhang X, Tunnell JW (2010). Experimental validation of the effects of microvasculature pigment packaging on in vivo diffuse reflectance spectroscopy. Lasers Surg. Med..

[25] Flock ST, Patterson MS, Wilson BC, Wyman DR (1989). Monte carlo modeling of light propagation in highly scattering tissues- I: model predictions and comparison with diffusion theory. IEEE Trans. Biomed. Eng..

[26] Amelink A, Robinson DJ, Sterenborg HJ (2008). Confidence intervals on fit parameters derived from optical reflectance spectroscopy measurements. J. Biomed. Opt..

[27] Roberts DW (2012). Glioblastoma multiforme treatment with clinical trials for surgical resection (aminolevulinic acid). Neurosurg. Clin. N. Am..

[28] Fan X, Ji S, Hartov A, Roberts DW, Paulsen KD (2014). Stereovision to MR image registration for cortical surface displacement mapping to enhance image-guided neurosurgery. Med. Phys..

